# Polygenic risk scores for late smoking initiation associated with the risk of schizophrenia

**DOI:** 10.1038/s41537-020-00126-z

**Published:** 2020-11-23

**Authors:** Kazutaka Ohi, Daisuke Nishizawa, Yukimasa Muto, Shunsuke Sugiyama, Junko Hasegawa, Midori Soda, Kiyoyuki Kitaichi, Ryota Hashimoto, Toshiki Shioiri, Kazutaka Ikeda

**Affiliations:** 1 Department of Psychiatry and Psychotherapy, Gifu University Graduate School of Medicine, Gifu, Japan; 2 Department of General Internal Medicine, Kanazawa Medical University, Ishikawa, Japan; 3 Addictive Substance Project, Tokyo Metropolitan Institute of Medical Science, Tokyo, Japan; 4 Department of Biomedical Pharmaceutics, Gifu Pharmaceutical University, Gifu, Japan; 5 Department of Pathology of Mental Diseases, National Institute of Mental Health, National Center of Neurology and Psychiatry, Kodaira, Tokyo, Japan; 6 Molecular Research Center for Children’s Mental Development, United Graduate School of Child Development, Osaka University, Suita, Osaka, Japan

**Keywords:** Genetics of the nervous system, Biomarkers, Schizophrenia

## Abstract

Patients with schizophrenia display characteristic smoking-related behaviors and genetic correlations between smoking behaviors and schizophrenia have been identified in European individuals. However, the genetic etiology of the association remains to be clarified. The present study investigated transethnic genetic overlaps between European-based smoking behaviors and the risk of Japanese schizophrenia by conducting polygenic risk score (PRS) analyses. Large-scale European genome-wide association study (GWAS) datasets (*n* = 24,114–74,035) related to four smoking-related intermediate phenotypes [(i) smoking initiation, (ii) age at smoking initiation, (iii) smoking quantity, and (iv) smoking cessation] were utilized as discovery samples. PRSs derived from these discovery GWASs were calculated for 332 Japanese subjects [schizophrenia patients, their unaffected first-degree relatives (FRs), and healthy controls (HCs)] as a target sample. Based on GWASs of European smoking phenotypes, we investigated the effects of PRSs on smoking phenotypes and the risk of schizophrenia in the Japanese population. Of the four smoking-related behaviors, the PRSs for age at smoking initiation in Europeans significantly predicted the age at smoking initiation (*R*^2^ = 0.049, *p* = 0.026) and the PRSs for smoking cessation significantly predicted the smoking cessation (*R*^2^ = 0.092, *p* = 0.027) in Japanese ever-smokers. Furthermore, the PRSs related to age at smoking initiation in Europeans were higher in Japanese schizophrenia patients than in the HCs and those of the FRs were intermediate between those of patients with schizophrenia and those of the HCs (*R*^2^ = 0.015, *p* = 0.015). In our target subjects, patients with schizophrenia had a higher mean age at smoking initiation (*p* = 0.018) and rate of daily smoking initiation after age 20 years (*p* = 0.023) compared with the HCs. A total of 60.6% of the patients started to smoke before the onset of schizophrenia. These findings suggest that genetic factors affecting late smoking initiation are associated with the risk of schizophrenia.

## Introduction

The lifespan of patients with schizophrenia is 10–20 years shorter than that of the general population^1–3^. Patients with schizophrenia have increased mortality and morbidity, mainly due to metabolic syndrome and premature cardiovascular disease^1–3^. A high rate of cigarette smoking contributes to metabolic syndrome and premature cardiovascular disease^3,4^. The prevalence of cigarette smoking in patients with schizophrenia is estimated to be two- to fivefold higher than in the general population worldwide^5–7^. Smokers with schizophrenia are generally “heavy smokers” who smoke more cigarettes and consume relatively larger total cigarette volumes^8^; these individuals generally experience nicotine dependence^9^.

The self-medication hypothesis argues that patients with schizophrenia smoke to alleviate their psychiatric symptoms, cognitive impairments, and/or antipsychotic-induced side effects^10,11^. In contrast, smoking itself may be a causal factor for the onset of schizophrenia^12^. Patients with schizophrenia display a higher risk of starting daily smoking at least 5 years before the onset of schizophrenia and starting psychiatric medication^13–15^, suggesting that the association between smoking and schizophrenia cannot be explained by the onset of illness or the effects of medication. Furthermore, the age at initiation of daily smoking is higher after age 20 years in individuals who later develop schizophrenia^13–15^. A drastic greater exposure for a brief period despite later age at smoking initiation may be at a higher risk of schizophrenia. Therefore, patients with schizophrenia display characteristic smoking-related behaviors: a high smoking initiation rate, later age at smoking initiation, high smoking quantity, and low smoking cessation rate.

Schizophrenia and smoking-related behaviors are highly heritable, with an estimated heritability of ~50–80%^16–18^. The risks of the onset of schizophrenia and smoking-related behaviors are increased in unaffected first-degree relatives (FRs) of patients with schizophrenia compared with healthy controls (HCs)^19–21^, suggesting that the relationships between schizophrenia and smoking-related behaviors are largely mediated by shared genetic factors. The Schizophrenia Working Group of the Psychiatric Genomics Consortium (PGC) and the Tobacco and Genetics (TAG) consortium have performed large-scale genome-wide association studies (GWASs) in samples of mainly European ancestry, to find risk genes for schizophrenia and smoking-related behaviors^16,22^. The TAG consortium has examined four elements of smoking behavior: smoking initiation, age at smoking initiation, smoking quantity, and smoking cessation. The GWAS from the PGC has identified 108 distinct genomic loci related to the risk of schizophrenia^22^. In contrast, among the GWASs of smoking-related phenotypes from the TAG, only an association between a genomic locus on 15q25 and smoking quantity has been revealed in smoking GWASs^16^. We found that genome-wide significant single-nucleotide polymorphisms (SNPs) on 15q25 shared between schizophrenia and smoking quantity contributed to a common pathophysiology underlying these phenotypes via altered *CHRNA5* expression in the brain^23^.

On the other hand, the risks of schizophrenia and smoking-related behaviors are mediated by not only genome-wide significant SNPs but also a polygenic component comprising the additive effects of a large number of common SNPs with weak effects^22,24–30^. Consistent with the comorbidity of schizophrenia and cigarette smoking behaviors, genetic correlations between the risk of schizophrenia, and higher smoking initiation, later age at smoking initiation and greater smoking quantity have been indicated by linkage disequilibrium score regression (LDSC) and polygenic risk score (PRS) analyses using European-based GWASs from the PGC and TAG^27,28,31^. However, these genetic correlations are restricted to findings derived from the same European-based GWAS datasets. To the best of our knowledge, it is unknown whether PRSs for European-based smoking behaviors are associated with the risk of schizophrenia in independent individuals of non-European ancestry.

As cigarette smoking causes severe health impairments in patients with schizophrenia, understanding the genetic basis underlying the comorbidity is clinically important. We hypothesized that genetic variants related to smoking behaviors in Europeans would transethnically overlap with genetic risk variants in Japanese schizophrenia patients and unaffected FRs. PRS analyses can examine whether the PRSs related to discovery GWASs can predict the risk of the phenotype in an independent target GWAS sample. The present study investigated the effects of PRSs based on GWASs of four European-based smoking-related intermediate phenotypes [(i) smoking initiation, (ii) age at smoking initiation, (iii) smoking quantity, and (iv) smoking cessation] on smoking behaviors and the risk of schizophrenia in the Japanese population by PRS analyses as well.

## Results

### Effects of PRSs for European-based smoking-related intermediate phenotypes on smoking behaviors in a Japanese population

**Fig. 1:**
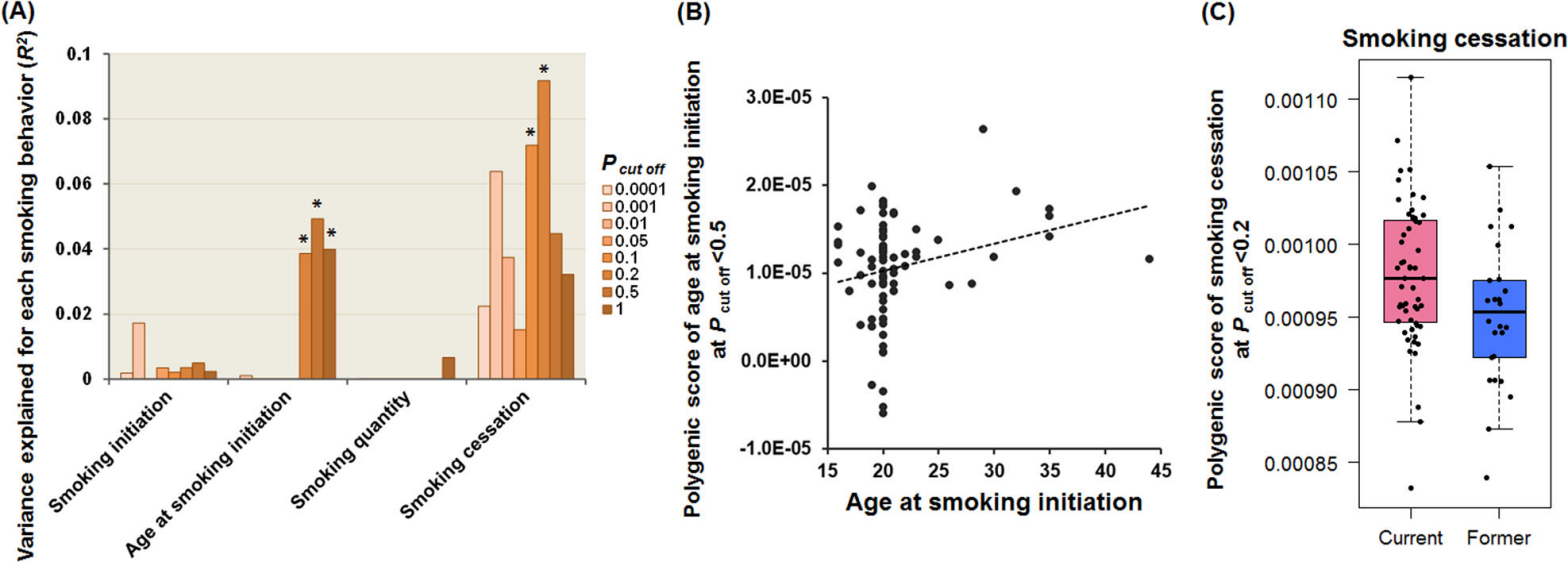
Effects of polygenic risk scores (PRSs) for European-based smoking-related intermediate phenotypes on smoking behaviors in a Japanese population. Effects of PRSs for four European-based smoking-related intermediate phenotypes based on each threshold (*P*_T cutoff_) on each smoking-related intermediate phenotype in Japanese target subjects (**a**). The y-axis shows the adjusted *R*^2^ or Nagelkerke’s pseudo-*R*^2^, indicating the explanatory power of the model. *p < 0.05. Positive correlation between PRSs related to European-based age at smoking initiation and age at smoking initiation in the target ever-smokers (**b**). Differences in PRSs based on European-based smoking cessation between Japanese current and former smokers (**c**). Box plots are used to visually show the distribution of PRSs. The median is shown by the center line that divides the box into two parts. Lower and upper bounds of box represent first and third quartiles, respectively. The lower and upper whiskers represent scores outside the middle 50%.

We first investigated the transethnic effect of PRSs for European-based smoking-related intermediate phenotypes (smoking initiation, age at smoking initiation, smoking quantity, and smoking cessation) on each smoking-related intermediate phenotype in Japanese target subjects at different *P*_T_ levels, respectively (Fig. 1a). Of four smoking-related intermediate phenotypes, the PRSs for European-based age at smoking initiation significantly predicted age at smoking initiation in Japanese ever-smokers (*n* = 81) (Fig. 1b, a maximum at *P*_T_ ≤ 0.5: adjusted *R*^2^ = 0.049, *p* = 0.026). In contrast, the PRSs for European-based smoking cessation were significantly and unexpectedly higher in Japanese current smokers (*n* = 55) than in former smokers (*n* = 26) (Fig. 1c, a maximum at *P*_T_ ≤ 0.2: Nagelkerke’s *R*^2^ = 0.092, *p* = 0.027). Even after including diagnostic status, age, and sex as covariates, these associations were still significant (age at smoking initiation, *p* = 0.043; smoking cessation, *p* = 0.013). There were no significant associations between the PRSs for European-based smoking initiation or smoking quantity and these smoking behaviors in a Japanese population, respectively (Fig. 1a, *p* > 0.05).

### Effects of PRSs for European-based smoking-related intermediate phenotypes on the risk of schizophrenia in a Japanese population

**Fig. 2:**
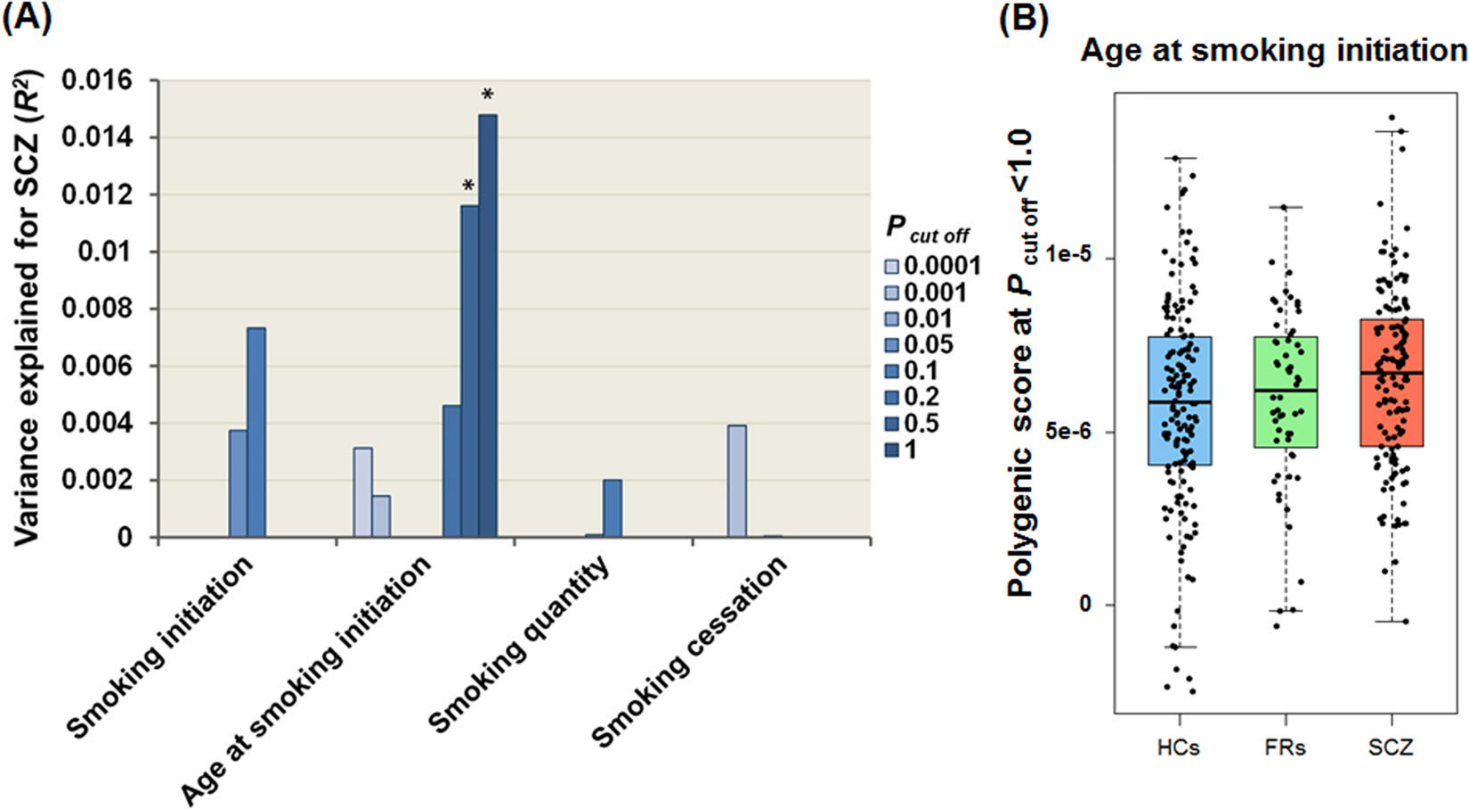
Effects of PRSs for European-based smoking-related intermediate phenotypes on the risk of SCZ in a Japanese population. Effects of PRSs for four European-based smoking-related intermediate phenotypes on the risk of SCZ (HCs, FRs, and patients with SCZ) (**a**). *p < 0.05. Differences in PRSs based on European-based age at smoking initiation among HCs, FRs, and patients with SCZ (**b**). HC healthy control; FR first-degree relative; SCZ schizophrenia.

To reveal genetic correlations between European-based smoking phenotypes and the risk of schizophrenia in a Japanese population, we investigated the effects of PRSs based on European-based smoking GWASs on risk levels in Japanese schizophrenia patients (schizophrenia patients vs. FRs vs. HCs) at different *P*_T cutoff_ levels (Fig. 2). The PRSs obtained from European-based GWAS for the age at smoking initiation were significantly different among Japanese HCs, FRs, and patients with schizophrenia (Fig. 2a, a maximum at *P*_T_ ≤ 1.0: adjusted *R*^2^ = 0.015, *p* = 0.015). The PRSs related to age at smoking initiation in Europeans were higher in Japanese patients with schizophrenia than in HCs and those in FRs were intermediate between those in patients with schizophrenia and those in HCs (Fig. 2b). In contrast, there were no significant differences in the PRSs obtained from European-based GWAS for the other three smoking-related intermediate phenotypes (smoking initiation, smoking quantity, or smoking cessation) among the diagnostic groups (Fig. 2a, *p* > 0.05). Even after excluding FRs, PRSs related to the European age at smoking initiation were significantly higher in Japanese patients with schizophrenia than in HCs (Supplementary Fig. 1, a maximum at *P*_T_ ≤ 1.0: Nagelkerke’s *R*^2^ = 0.028, *p* = 0.017). The directions of these associations between patients with schizophrenia vs. FRs vs. HCs and patients with schizophrenia vs. HCs were identical. There was no significant interaction between the PRSs for age at smoking initiation and ever/never smoking status with regard to the risk of schizophrenia in a Japanese population (Supplementary Fig. 2, *p* = 0.88).

### Differences in age at smoking initiation among Japanese HCs, FRs, and patients with schizophrenia

**Fig. 3:**
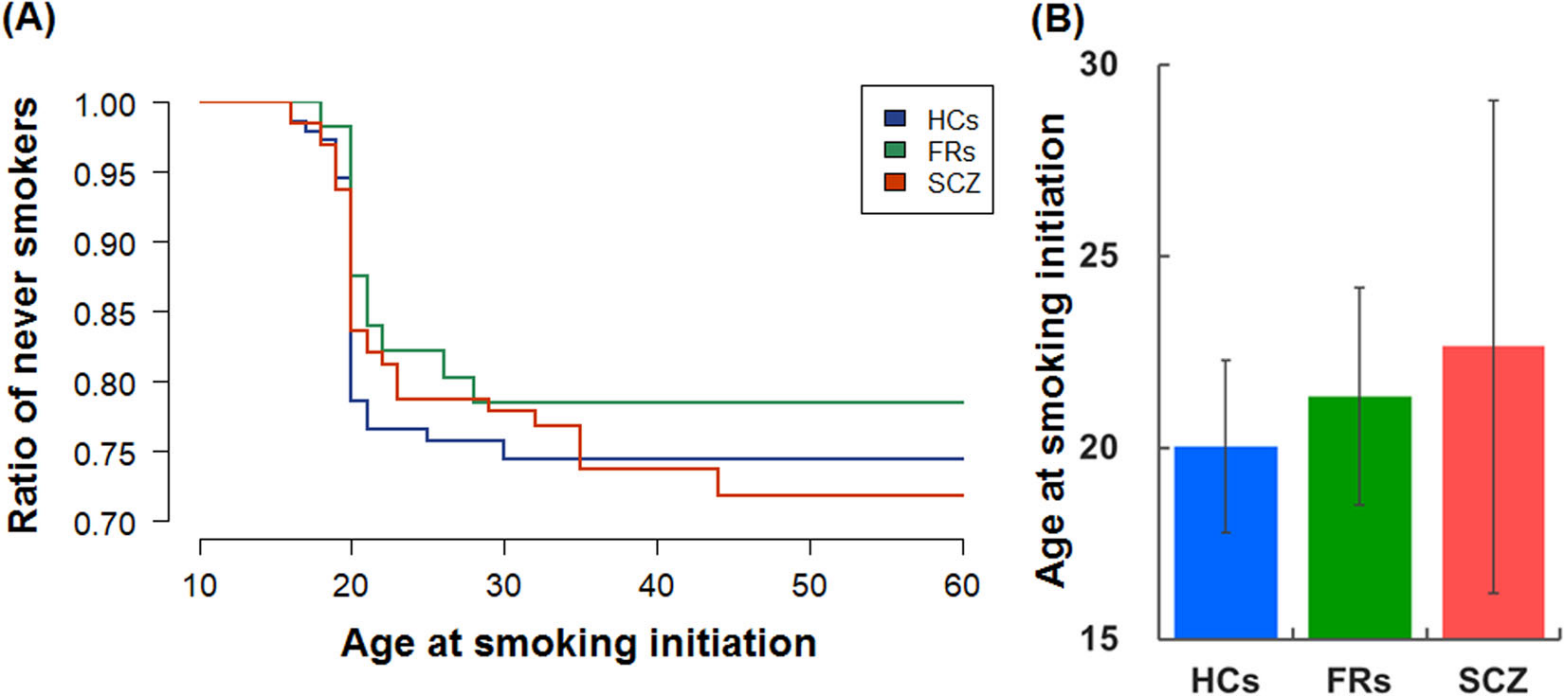
Differences in age at smoking initiation among Japanese HCs, FRs, and patients with SCZ. A survival analysis of onset age for daily smoking among Japanese HCs, FRs, and patients with SCZ (**a**). Differences in the mean ages at smoking initiation among the diagnostic groups in ever-smokers (**b**).

We performed a survival analysis of the age at onset for daily smoking (conversion into ever-smoker from never-smoker) among Japanese HCs, FRs, and patients with schizophrenia (Fig. 3a), but the survival curves of the age at onset for daily smoking were not significantly different among the diagnostic groups (*p* = 0.70). In contrast, the mean ages at smoking initiation were significantly different among the diagnostic groups in ever-smokers (Fig. 3b, *β* = 0.26, *p* = 0.018). Even after including age and sex as covariates, differences in the mean ages at smoking initiation were still significant (*β* = 0.29, *p* = 0.013). Patients with schizophrenia showed a higher age at smoking initiation than the other groups. As schizophrenia is associated with a higher rate of smoking initiation after age 20 years (>20) in multiple ethnic populations^13–15^, we investigated the rate of smoking initiation after age 20 years among HCs, FRs, and patients with schizophrenia. The rates of smoking initiation after age 20 years, with age and sex as covariates, were significantly different among the diagnostic groups (*p* = 0.023). The rates of smoking initiation after age 20 years were 13.9% (5/36) in HCs, 41.7% (5/12) in FRs, and 36.4% (12/33) in patients with schizophrenia. Many smokers who were HCs started to smoke at age 20 years, whereas smokers who were FRs and patients with schizophrenia gradually started to smoke after age 20 years. Furthermore, after age 20 years, smoking initiation rates have been higher in overall schizophrenia patients and in schizophrenia patients who started daily smoking at least 5 years before the onset of the disorder compared with HCs or general population^13–15^. Histograms of the age at smoking initiation, age at onset of schizophrenia, and the age at smoking initiation minus the age at the onset of schizophrenia in our target patients are shown in Supplementary Fig. 3. Of 33 schizophrenia patients who had ever smoked, 60.6% started to smoke before the onset of the disorder, 3.0% started to smoke at the onset of the disorder, and 36.4% started to smoke after the onset of the illness.

## Discussion

The present study examined for the first time whether European-based PRSs for smoking-related intermediate phenotypes (smoking initiation, age at smoking initiation, smoking quantity, and smoking cessation) based on large-scale GWASs from the TAG consortium transethnically affect smoking behaviors and the risk of schizophrenia in a Japanese population by PRS analyses. Of the four smoking-related behaviors, the PRSs for European-based age at smoking initiation and smoking cessation significantly predicted age at smoking initiation and smoking cessation, respectively, in Japanese ever-smokers. Furthermore, the PRSs related to European-based age at smoking initiation were linearly associated with the risk of schizophrenia in the Japanese population. Among Japanese ever-smokers, patients with schizophrenia had a delayed mean age at smoking initiation, especially after age 20 years, but most patients started to smoke before the onset of the disorder.

Consistent with the positive genetic correlation between the risk of schizophrenia and age at smoking initiation in individuals of European ancestry according to LDSC analysis^28^, the PRSs related to age at smoking initiation in Europeans predicted the risk of schizophrenia in non-European subjects regardless of the differences in sample ethnicity and analytical approach (LDSC and PRS analyses). Considering that earlier age at smoking initiation contributes to a higher risk of nicotine dependence in the general population^32^, it is expected that genetic factors for age at smoking initiation would be earlier (lower) in patients with schizophrenia than in HCs. However, age at smoking initiation in patients with schizophrenia was genetically and clinically later compared with that in HCs. The PRSs related to age at smoking initiation explained 1.5% of the variance in schizophrenia status. Furthermore, even after including confounding factors, such as age, sex, and education years, as covariates, the PRSs related to age at smoking initiation were strongly associated with the diagnostic status (*p* = 5.98 × 10^−3^). We suggest that smoking behaviors, i.e., later age at smoking initiation but initiation before age at onset of the disorder, genetically contribute to the risk of developing schizophrenia.

As unaffected FRs share approximately half of the genetic risk of schizophrenia, PRSs for schizophrenia in unaffected FRs are intermediate between those of schizophrenia patients and HCs^29,33,34^. In addition to this evidence, we found that the PRSs for age at smoking initiation were higher in schizophrenia patients than in HCs, and those in FRs were intermediate between those in patients with schizophrenia and those in HCs. These findings support the concept that there are shared genetic components between the risk of schizophrenia and age at smoking initiation. In contrast, it may also be a causal effect in either direction. To explore the causal effect, further study using Mendelian randomization is warranted.

Despite positive genetic correlations between the risk of schizophrenia and smoking initiation and smoking quantity in individuals of European ancestry according to LDSC analysis^27,28,31^, we could not find genetic overlaps in the Japanese population. This might be the result of socioenvironmental changes surrounding smoking in Japan that occurred prior to ~2010. Governmental health insurance has approved smoking cessation treatment in Japan since 2006 and the price per pack of cigarettes increased >30% in 2010. Furthermore, cigarette smoking was gradually restricted in public places and in psychiatric hospitals in Japan. These events may have affected the decline in the rate of ever-smokers and smoking quantity since 2010.

The PRSs for European-based smoking initiation and heaviness did not significantly predict smoking initiation or heaviness in Japanese population. The PRSs for European-based smoking cessation were unexpectedly higher in Japanese current smokers than in former smokers. The reasons why we could not detect significant transethnic effects of PRSs for European-based smoking-related intermediate phenotypes on Japanese smoking behaviors might be a power issue of our samples. In contract, the initiation PRSs were much more associated with risk taking/impulsivity and make more or less risky initiation, and might be social differences in Japan. Therefore, we might not have predicted the association by the European score. Given that these European-based PRSs did not predict Japanese smoking behaviors, it was not surprising that they did not predict risk of schizophrenia.

Cigarettes are very refined tools to deliver nicotine to the brain. Cigarette smoking modulates dopaminergic activity in the brain through inducing the release and inhibiting the degradation^35^. Furthermore, cigarette smoking can reduce impairments associated with dopamine hypofunction in the prefrontal cortex^35^. Therefore, patients with schizophrenia may practice self-medication behavior to alleviate their psychiatric symptoms, cognitive impairments, and/or antipsychotic-induced side effects^10,11^. Despite our evidence that patients with schizophrenia initiate smoking at a later age, patients may develop nicotine dependence through self-medicating behavior and as a result of genetic factors affecting their susceptibility to nicotine dependence.

There are some limitations of the interpretations of our findings. Recently, the GWAS and Sequencing Consortium of Alcohol and Nicotine use has performed larger-scale GWAS for smoking-related behaviors in up to 1.2 million individuals^36^. A critical factor in determining if the polygenic components can explain a target trait in independent participants is the sample size of the discovery GWAS^37,38^. Therefore, further study using the latest and largest-scale GWAS is required to confirm our findings. Our target sample size might have been insufficient to identify possible associations between the risk of schizophrenia and PRSs related to the other three smoking-related behaviors. Furthermore, the sample size of Japanese ever-smokers in our target cohort was relatively small. Therefore, negative findings should be interpreted with cation, as our sample size was small for PRS analysis. Further investigation using a larger sample is warranted. Compared with the mean ages of the FRs and patients with schizophrenia, the mean age of the HCs was younger (37.2 ± 14.1 years), potentially including future ever-smokers as never smokers. However, the possibility that HCs have a risk of smoking initiation may be low, because the mean age at smoking initiation in HCs was 20.0 ± 2.2 years.

In conclusion, the common polygenic factors for European-based age at smoking initiation, which is associated with age at smoking initiation in Japanese ever-smokers, could transethnically explain susceptibility to schizophrenia in a Japanese population. These findings suggest that there are common transethnic genetic factors for the risk of schizophrenia and delayed age at smoking initiation between individuals of European and non-European ancestry. As smoking behavior is a protectable and treatable cause of morbidity and mortality in schizophrenia, as well as the onset of other illnesses, understanding the genetic etiology underlying smoking behavior is important.

## Methods

### Discovery European samples

Several publicly available smoking-related GWAS datasets [(i) smoking initiation, (ii) age at smoking initiation, (iii) smoking quantity, and (iv) smoking cessation (https://www.med.unc.edu/pgc/results-and-downloads)] from individuals of European ancestry from the TAG Consortium^16^ were used as discovery samples to identify variants related to each intermediate smoking phenotype and their *p*-values and effect sizes (*β*).

The four smoking-related intermediate phenotypes were defined as follows^16^: (i) smoking initiation was assessed as ever vs. never regular smokers. Regular smokers and never regular smokers were defined as those who reported having smoked ≥100 cigarettes and those who reported having smoked between 0 and 99 cigarettes during their lifetime, respectively. (ii) Age at smoking initiation in ever-smokers was assessed as the age at which the individual first tried smoking cigarettes or began smoking cigarettes regularly. The age at smoking initiation was transformed using the natural logarithm. (iii) Smoking quantity in ever-smokers was assessed as the average or maximum number of cigarettes smoked per day (CPD). (iv) Smoking cessation in ever-smokers was assessed as former vs. current smokers. Current smokers reported that they smoked at the time of the interview, whereas former smokers had quit smoking at least 1 year before the interview. Smokers who had quit smoking for <1 year at interview were excluded, because relapse is the most common in the first year of quitting smoking.

A detailed description of the sample collection, sample information, genotyping, quality control (QC), and imputation procedures applied in the discovery samples has been described previously^16^. Briefly, the 16 TAG studies involved genotyping on six different GWAS platforms and performed their own QC and imputation. Only subjects of European ancestry were included. Genotype imputation was performed using the HapMap-2 Utah residents with Northern and Western European ancestry (CEU) samples as a reference panel. Genotype imputation resulted in a common set of ~2.5 million genetic variants. Imputed allele dosages for each genetic variant were tested for associations with each intermediate smoking phenotype under additive logistic [(i) smoking initiation and (iv) smoking cessation] or linear [(ii) age at smoking initiation and (iii) smoking quantity] regression models. Case–control status was adjusted if the primary study was case–control in design. These analyses were performed separately for males and females. A fixed-effect meta-analysis was performed for each intermediate smoking phenotype. The sample sizes for each GWAS (Table 1) and the number of SNPs used for the PRS analyses at each liberal significance threshold (*P*_T cutoff_) (Supplementary Table 1) are also provided.

**Table 1:** Sample sizes for European-based discovery GWASs of four smoking-related intermediate phenotypes.

Smoking phenotype	Description	Authors (year)	GWS locus	Sample sizes (*n*)
Smoking initiation	Ever vs. never smokers	Furberg et al.^16^	0	74,035
Age at smoking initiation	Age at started smoking cigarettes	Furberg et al.^16^	0	24,114
Smoking quantity	Number of cigarettes smoked per day	Furberg et al.^16^	1	38,181
Smoking cessation	Former vs. current smokers	Furberg et al.^16^	0	41,278

*GWS* genome-wide significant.

### Target Japanese sample

The target sample (*n* = 332) comprised 130 patients with schizophrenia (50 males/80 females, mean age ± SD: 42.9 ± 13.1 years), 56 of their unaffected FRs (40 parents/12 siblings/4 offspring, 18 males/38 females, 56.8 ± 15.6 years), and 146 HCs (97 males/49 females, 37.2 ± 14.1 years). All of these subjects participated in our previous study^29^. The study sample was recruited from the Schizophrenia Non-Affected Relative Project^29,39^. All subjects were of Japanese descent and had no biological first- or second-degree relatives within their own diagnostic groups. A detailed description of sample recruitment and diagnosis has been provided previously^7,29,39–43^. Briefly, each patient was diagnosed on the basis of unstructured clinical interviews, medical records, and clinical conferences according to the criteria in the fifth edition of the Diagnostic and Statistical Manual of Mental Disorders (DSM-5). FRs and HCs were evaluated using the Structured Clinical Interview for DSM-IV-Non-Patient version. Written informed consent was obtained from all participants after the procedures had been thoroughly explained. This study was performed in accordance with the World Medical Association’s Declaration of Helsinki and was approved by the Research Ethics Committees of Gifu University and Kanazawa Medical University.

The definitions of the four smoking-related intermediate phenotypes in the target sample were as follows: (i) smoking initiation was assessed as ever vs. never regular smokers. (ii) Age at smoking initiation in ever-smokers was assessed as the age at which the individual first tried smoking cigarettes. (iii) Smoking quantity in ever-smokers was assessed as the average number of CPDs. (iv) Smoking cessation in ever-smokers was assessed as former vs. current smokers. Current smokers reported that they smoked at the time of the interview, whereas former smokers had quit smoking before the interview. These definitions in the current study were not strict compared with the original GWAS^16^, but these definitions were similar between studies.

A detailed description of the genotyping, QC and imputation procedures applied in the target sample has been provided previously^29^. Briefly, venous blood was collected from the target subjects and genomic DNA was extracted from the whole blood samples. Genotyping was performed using the Infinium OmniExpressExome-8 v1.4 BeadChip (Illumina, San Diego, CA, USA). After applying QC, including the genotype call rate, genotype call frequency, sex chromosome anomalies, and sample relatedness in each diagnostic group, 332 individuals were included in the current study. The demographic information of the diagnostic groups is summarized in Table 2. Furthermore, after the exclusion of SNPs that were duplicated or ambiguous, were localized on the Y chromosome or mitochondria, deviated from Hardy–Weinberg equilibrium, or had a low minor allele frequency, 627,789 SNPs were retained. Genotype imputation was performed using the 1000 Genomes Project Phase 3 dataset [https://mathgen.stats.ox.ac.uk/impute/1000GP_Phase3.html]^44^ as a reference panel. For the PRS analysis, SNPs with high imputation quality (>0.9) were retained. Finally, 8,339,066 variants were considered in the PRS analysis.

**Table 2:** Demographic information for the Japanese target GWAS sample with SCZ.

	HC	FR	SCZ	
Variables	(*n* = 146)	(*n* = 56)	(*n* = 130)	*p*-values (*F* or *χ*^*2*^)
Age (years)	37.2 ± 14.1	56.8 ± 15.6	42.9 ± 13.1	2.49 × 10^−21^ (55.0)
Sex (male/female)	97/49	18/38	50/80	3.22 × 10^−7^ (30.0)^a^
Education (years)	16.1 ± 2.4	12.8 ± 2.1	12.6 ± 2.2	2.05 × 10^−32^ (91.8)
Estimated premorbid IQ	108.5 ± 7.7	99.7 ± 9.1	98.7 ± 10.4	5.38 × 10^−18^ (45.0)
Ever/never smoked	36/110	12/44	33/97	0.83 (0.3)
Age at smoking initiation^b^	20.0 ± 2.2	21.3 ± 2.8	22.6 ± 6.4	0.062 (2.9)
Cigarettes per day^b^	16.4 ± 10.3	19.0 ± 14.4	21.3 ± 14.3	0.29 (1.3)
Current/former smoker^b^	24/12	2/10	29/4	3.50 × 10^−5^ (20.5)^a^
CPZ-eq. (mg/day)	0	0	509.6 ± 512.7	–
Age at onset (years)	–	–	26.9 ± 10.6	–
DOI (years)	–	–	15.8 ± 11.3	–
PANSS-positive symptoms	–	–	16.0 ± 6.2	–
PANSS-negative symptoms	–	–	17.8 ± 6.8	–

*CPZ-eq.* chlorpromazine equivalent of total antipsychotics, *DOI* duration of illness, *FR* first-degree relative, *HC* healthy control, *PANSS* Positive and Negative Syndrome Scale, *SCZ* schizophrenia. The mean ± SD and *p*-values are shown. The significant *p*-values (*p* < 0.05) are shown in boldface and underlined. Complete demographic information was not obtained for all subjects (estimated premorbid IQ in HCs, *n* = 145). Co*n*tinuous variables and categorical variables were analyzed using parametric analysis of variance (ANOVA) and Pearson’s *χ*^2^-test, respectively.

^a^*χ*^2^-test.

^b^Calculated among ever regular smokers (HC, *n* = 36; FR, *n* = 12; SCZ, *n* = 33).

### Statistical analyses

The SNPs in the target sample were pruned based on a pairwise *r*^2^ threshold of 0.25 and a window size of 200 SNPs to remove SNPs that were in linkage disequilibrium^29^. After pruning, 1,354,311 independent SNPs remained. PRSs constructed from SNPs showing a nominal association with each smoking-related phenotype in the discovery GWASs according to the following eight *P*_T cutoff_ values were then calculated: *P*_T_ ≤ 0.0001, *P*_T_ ≤ 0.001, *P*_T_ ≤ 0.01, *P*_T_ ≤ 0.05, *P*_T_ ≤ 0.1, *P*_T_ ≤ 0.2, *P*_T_ ≤ 0.5, and *P*_T_ ≤ 1. For each individual included in the target sample, a PRS was calculated by weighting the scores for the “risk SNPs” by the logarithm of the odds ratio (OR) converted from the *β* (logOR) observed in each smoking-related discovery dataset. The score, consisting of the number of risk alleles (0, 1, or 2) multiplied by the logOR, was summed over all of the SNPs in the *P*_T_-SNP sets for each individual in the target sample. To examine the effects of the European PRSs based on each *P*_T cutoff_ on smoking behaviors and the risk of schizophrenia in the Japanese sample population, we used R 3.6.1 (http://www.r-project.org/) to perform linear regression or logistic regression with diagnostic status (schizophrenia patients, FRs, and HCs) or each smoking-related phenotype as a dependent variable and each PRS based on each European smoking GWAS as the independent variable. We compared the differences in the adjusted *R*^2^ for linear regression or Nagelkerke’s pseudo-*R*^2^ for logistic regression, which are a measure of the variance explained by the model. A survival analysis of age at onset for daily smoking among HCs, FRs and patients with schizophrenia was performed using R. The PRSs at each *P*_T cutoff_ were highly correlated with each other and were not independent. Furthermore, the smoking-related intermediate phenotypes were also marginally correlated with each other and were not independent. Therefore, the *p*-values based on different *P*_T cutoff_ values were not corrected and the significance level for the current study was set at *p* < 0.05.

### Reporting summary

Further information on research design is available in the Nature Research Reporting Summary linked to this article.

## Supplementary information

Supplementary Information

Reporting Summary

## Data Availability

The discovery data are publicly available (https://www.med.unc.edu/pgc/results-and-downloads). Our target data are not publicly available due to them containing information that could compromise research participant privacy/consent.
