# Supplementary material for: Polygenic risk scores for late smoking initiation associated with the risk of schizophrenia
Source: NPJ Schizophr. 2020 Nov 23;6:36. doi: 10.1038/s41537-020-00126-z (PMC7684279; doi:10.1038/s41537-020-00126-z)

Supplementary Information

Supplementary Table 1 Number of SNPs used for PRS analyses at each $P_{T\ cutoff}$ in the Japanese target sample

Smoking phenotype	Discovery SNPs (n)	Number of SNPs used for analyses at P_T in target sample 0.0001	0.001	0.01	0.05	0.1	0.2	0.5	1
**Smoking initiation**	2,455,846	14	142	1,237	5,534	10,602	20,443	48,827	95,292
**Age at smoking initiation**	2,457,545	11	90	992	4,915	9,831	19,435	47,842	95,336
**Smoking quantity**	2,459,118	19	135	1,058	5,057	10,069	19,795	48,483	95,505
**Smoking cessation**	2,456,554	10	97	1,058	5,083	9,997	19,622	48,212	95,275

Supplementary Figure 1 Effects of PRSs for four European-based smoking-related intermediate phenotypes based on each threshold ($P_{T\ cutoff}$) on the risk of Japanese SCZ between HCs and patients with SCZ **(A)**. The y-axis shows Nagelkerke's pseudo-R^2, indicating the explanatory power of the model. HC, healthy control; SCZ, schizophrenia. $^*p<0.05$.

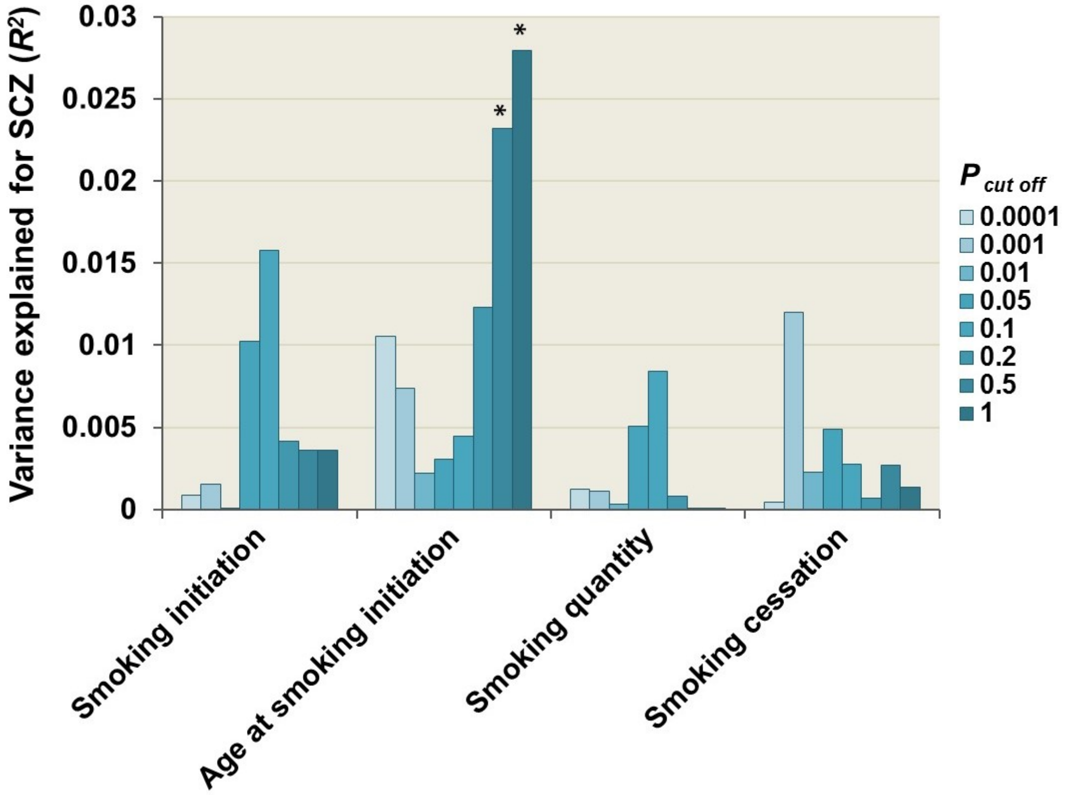

Supplementary Figure 2 Impact of interaction between PRSs related to European-based age at smoking initiation and ever/never smoker status on the risk of Japanese SCZ. There was no significant interaction between the PRSs and ever/never smoker status on the diagnostic status (*p*=0.88).

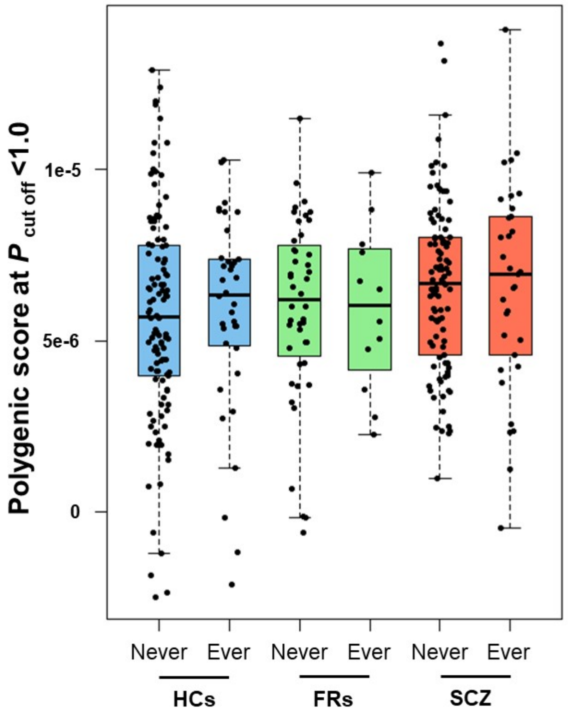

Supplementary Figure 3 Age at smoking initiation and age at onset of SCZ. Histograms of age at smoking initiation and age at onset of SCZ **(A)**. A histogram of the age at smoking initiation minus the age at the onset of SCZ **(B)**.

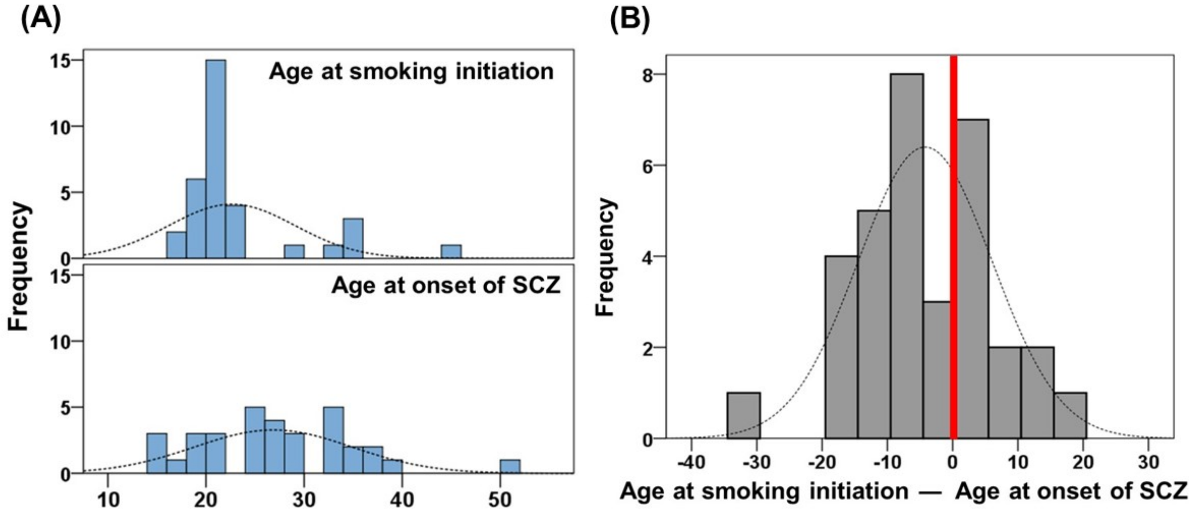

Supplement: Supplementary file 1 — Supplementary Information [file 41537_2020_126_MOESM1_ESM.pdf]
