# Supplementary material for: Polygenic risk scores for late smoking initiation associated with the risk of schizophrenia
Source: NPJ Schizophr. 2020 Nov 23;6:36. doi: 10.1038/s41537-020-00126-z (PMC7684279; doi:10.1038/s41537-020-00126-z)
Supplement: Supplementary file 2 — Reporting Summary [file 41537_2020_126_MOESM2_ESM.pdf]

nature research

Corresponding author(s): Kazutaka Ohi

Last updated by author(s): Jul 24, 2020

Reporting Summary

Nature Research wishes to improve the reproducibility of the work that we publish. This form provides structure for consistency and transparency in reporting. For further information on Nature Research policies, see our Editorial Policies and the Editorial Policy Checklist.

Statistics

For all statistical analyses, confirm that the following items are present in the figure legend, table legend, main text, or Methods section.

n/a	Confirmed	
☐	☒	The exact sample size (*n*) for each experimental group/condition, given as a discrete number and unit of measurement
☐	☒	A statement on whether measurements were taken from distinct samples or whether the same sample was measured repeatedly
☐	☒	The statistical test(s) used AND whether they are one- or two-sided<br>*Only common tests should be described solely by name; describe more complex techniques in the Methods section.*
☐	☒	A description of all covariates tested
☐	☒	A description of any assumptions or corrections, such as tests of normality and adjustment for multiple comparisons
☐	☒	A full description of the statistical parameters including central tendency (e.g. means) or other basic estimates (e.g. regression coefficient) AND variation (e.g. standard deviation) or associated estimates of uncertainty (e.g. confidence intervals)
☐	☒	For null hypothesis testing, the test statistic (e.g. *F*, *t*, *r*) with confidence intervals, effect sizes, degrees of freedom and *P* value noted<br>*Give P values as exact values whenever suitable.*
☒	☐	For Bayesian analysis, information on the choice of priors and Markov chain Monte Carlo settings
☒	☐	For hierarchical and complex designs, identification of the appropriate level for tests and full reporting of outcomes
☐	☒	Estimates of effect sizes (e.g. Cohen's *d*, Pearson's *r*), indicating how they were calculated

Our web collection on statistics for biologists contains articles on many of the points above.

Software and code

Policy information about availability of computer code

Data collection	Several publicly available smoking-related GWAS datasets [(i) smoking initiation, (ii) age at smoking initiation, (iii) smoking quantity, and (iv) smoking cessation (https://www.med.unc.edu/pgc/results-and-downloads)] from individuals of European ancestry from the TAG Consortium were used as discovery samples.
Data analysis	Plink 1.9, R 3.6.1

For manuscripts utilizing custom algorithms or software that are central to the research but not yet described in published literature, software must be made available to editors and reviewers. We strongly encourage code deposition in a community repository (e.g. GitHub). See the Nature Research guidelines for submitting code & software for further information.

Data

Policy information about availability of data

All manuscripts must include a data availability statement. This statement should provide the following information, where applicable:

- Accession codes, unique identifiers, or web links for publicly available datasets
- A list of figures that have associated raw data
- A description of any restrictions on data availability

Our data are not publicly available due to them containing information that could compromise research participant privacy/consent.

Field-specific reporting

Please select the one below that is the best fit for your research. If you are not sure, read the appropriate sections before making your selection.

☒ Life sciences ☐ Behavioural & social sciences ☐ Ecological, evolutionary & environmental sciences

For a reference copy of the document with all sections, see nature.com/documents/nr-reporting-summary-flat.pdf

Life sciences study design

All studies must disclose on these points even when the disclosure is negative.

Sample size	Based on previous studies, we selected our sample sizes.
Data exclusions	A detailed description of sample recruitment and diagnosis has been provided previously 7,29,33-37.
Replication	no replication data
Randomization	N/A
Blinding	N/A

Reporting for specific materials, systems and methods

We require information from authors about some types of materials, experimental systems and methods used in many studies. Here, indicate whether each material, system or method listed is relevant to your study. If you are not sure if a list item applies to your research, read the appropriate section before selecting a response.

Materials & experimental systems

n/a	Involved in the study	
☒	☐	Antibodies
☒	☐	Eukaryotic cell lines
☒	☐	Palaeontology and archaeology
☒	☐	Animals and other organisms
☐	☒	Human research participants
☒	☐	Clinical data
☒	☐	Dual use research of concern

Methods

n/a	Involved in the study	
☒	☐	ChIP-seq
☒	☐	Flow cytometry
☒	☐	MRI-based neuroimaging

Human research participants

Policy information about studies involving human research participants

Population characteristics	The target sample (n=332) comprised 130 patients with schizophrenia (50 males/80 females, mean age±SD: 42.9±13.1 years), 56 of their unaffected FRs (40 parents/12 siblings/4 offspring, 18 males/38 females, 56.8±15.6 years) and 146 HCs (97 males/49 females, 37.2±14.1 years).
Recruitment	The study sample was recruited from the Schizophrenia Non-Affected Relative Project (SNARP).
Ethics oversight	All subjects were of Japanese descent and had no biological first- or second-degree relatives within their own diagnostic groups.

Note that full information on the approval of the study protocol must also be provided in the manuscript.
